# *PSMC3* promotes RNAi by maintaining* AGO2* stability through *USP14*

**DOI:** 10.1186/s11658-022-00411-y

**Published:** 2022-12-17

**Authors:** Yan Jia, Jianing Zhao, Tao Yu, Xue Zhang, Xiaozhen Qi, Tongxin Hao, Zeyuan Jin, Xiaoqing Zhao

**Affiliations:** 1 Department of Pathogen Biology, School of Basic Medical Sciences, Tianjin Medical University, No. 22 Qi-Xiang- Tai Road, Tianjin, 300070 China; 2 Department of Clinical Laboratory, The Second Hospital of Shandong University, Jinan, Shandong China

**Keywords:** *AGO2*, *PSMC3*, *USP14*, deubiquitination, proteasome, RNAi

## Abstract

**Background:**

Argonaute 2 (*AGO2*), the only protein with catalytic activity in the human Argonaute family, is considered as a key component of RNA interference (RNAi) pathway. Here we performed a yeast two-hybrid screen using the human Argonaute 2 PIWI domain as bait to screen for new *AGO2*-interacting proteins and explored the specific mechanism through a series of molecular biology and biochemistry experiments.

**Methods:**

The yeast two-hybrid system was used to screen for *AGO2*-interacting proteins. Co-immunoprecipitation and immunofluorescence assays were used to further determine interactions and co-localization. Truncated plasmids were constructed to clarify the interaction domain. EGFP fluorescence assay was performed to determine the effect of *PSMC3* on RNAi. Regulation of *AGO2* protein expression and ubiquitination by *PSMC3* and *USP14* was examined by western blotting. RT-qPCR assays were applied to assess the level of *AGO2* mRNA. Rescue assays were also performed.

**Results:**

We identified *PSMC3* (proteasome 26S subunit, ATPase, 3) as a novel *AGO2* binding partner. Biochemical and bioinformatic analysis demonstrates that this interaction is performed in an RNA-independent manner and the N-terminal coiled-coil motif of *PSMC3* is required. Depletion of *PSMC3* impairs the activity of the targeted cleavage mediated by small RNAs. Further studies showed that depletion of *PSMC3* decreased *AGO2* protein amount, whereas *PSMC3 *overexpression increased the expression of *AGO2* at a post-translational level. Cycloheximide treatment indicated that* PSMC3* depletion resulted in a decrease in cytoplasmic *AGO2* amount due to an increase in* AGO2* protein turnover. The absence of *PSMC3* promoted ubiquitination of* AGO2*, resulting in its degradation by the 26S proteasome. Mechanistically, *PSMC3* assists in the interaction of *AGO2* with the deubiquitylase* USP14*(ubiquitin specific peptidase 14) and facilitates *USP14*-mediated deubiquitination of *AGO2*. As a result, *AGO2* is stabilized, which then promotes RNAi.

**Conclusion:**

Our findings demonstrate that *PSMC3* plays an essential role in regulating the stability of *AGO2* and thus in maintaining effective RNAi.

**Supplementary Information:**

The online version contains supplementary material available at 10.1186/s11658-022-00411-y.

## Background

The Argonaute proteins were first identified in plants [1] and are highly conserved across species, with many organisms encoding multiple family members [2]. Argonaute proteins can be subdivided phylogenetically into the AGO subfamily and the PIWI subfamily. Human AGO subfamily consists of four Argonaute genes (*AGO1*, *AGO2*, *AGO3*, and *AGO4*), which are ubiquitously expressed and associated with miRNAs and siRNAs[3, 4]. Although they all suppress translation of their target mRNAs, only* AGO2* has the slicer activity, and a catalytic triad consisting of Asp-Asp-His motif has been identified in this protein [5, 6]. *AGO2* consists of four functional core domains (N, PAZ, MID, and PIWI) and two domain linkers (L1 and L2) [7]. The analysis of the crystal structure of *AGO2*-bound pre-miRNAs showed the N domain initiates duplex RNA unwinding during RISC (RNA-induced silencing complex) assembly, the PAZ domain is an RNA binding module that specifically recognizes the 3′-protruding ends of the small RNAs, the MID domain harbors the 5′-phosphate of the guide RNAs, and the PIWI domain contains an RNaseH-like structure, which is necessary for mRNA cleavage [8–10]. Current studies on *AGO2* have shown that it can be involved not only in cytoplasmic RNAi, but also in gene regulatory processes in nuclei [11–13]. Moreover, *AGO2* has also been found to regulate other cellular processes, such as alternative polyadenylation, transposon repression, and translational activation [14–16]. In addition, direct regulation of the stemness genes by nuclear* AGO2* is also crucial for stem cell self-renewal, survival, and differentiation [17].

RNA interference depends on RNA-induced silencing complex to regulate gene expression. During RNAi, small RNAs are loaded into the complex and then the complex recognizes the target mRNA [18, 19]. Perfect complementarity between the small RNAs and the target mRNAs promotes endonucleolytic cleavage [20], whereas mismatches lead to suppression of gene expression through translational repression or mRNA deadenylation [21]. Given that *AGO2* is a core component and catalytic engine of RISC, it is essential for small-RNA-guided posttranscriptional gene silencing. Many proteins have been identified to be associated with *AGO2* and function in both RNAi and miRNA pathways[22–24]. The PIWI domain of human Argonaute proteins has been shown to contain an RNaseH-like structure, which is not only necessary for *AGO2*-mediated targeted mRNAs cleavage guided by small RNAs [7, 9], but is also involved in protein–protein interaction between Argonaute and *Dicer* [25], *TRBP* [26], and *GW182 *[27]. Here we identified *PSMC3* as a novel *AGO2*-interacting protein in a yeast two-hybrid screen using the PIWI domain of *AGO2* as bait. *PSMC3* is a multifunctional protein directly involved in the regulation of gene transcription as well as in protein degradation [28, 29]. We support a critical role of *PSMC3* in ubiquitin-mediated proteasomal degradation of* AGO2*, which further also impacts the targeted cleavage mediated by small RNAs.

## Methods

### Plasmids and oligos

The generation of constructs used in this study is detailed in Additional file 1: Experimental Procedures.

### Antibodies and reagents

Anti-HA, anti-Myc, anti-Flag, anti-GFP, and anti-ubiquitin primary antibodies were from Sigma-Aldrich (MO, USA). Immunoaffinity-purified rabbit polyclonal antibodies against *AGO2*, *PSMC3*, and *GAPDH* were from Saier Biotech (Tianjin, China). Horseradish-peroxidase-conjugated anti-mouse and anti-rabbit secondary antibodies were from Zhongshan Goldenbridge Biotechnology (Beijing, China). FITC/TRITC-conjugated secondary antibodies were purchased from Jackson Immunoresearch Laboratories (PA, USA).

MG132 was obtained from Calbiochem (Darmstadt, Germany), and chloroquine (CQ) and cycloheximide (CHX) were purchased from Sigma-Aldrich (MO, USA). Compounds were dissolved in DMSO and used at the concentrations indicated.

### Yeast two-hybrid screen

A Stratagene Cytotrap system human lung library (La Jolla, CA, USA) was screened according to the manufacturer’s instructions. Further details are provided in Additional file 1: Experimental Procedures.

### Cell culture and transfection

HeLa cells, Huh7 cells, and A549 cells were obtained from ATCC cell bank and kept by the laboratory. HeLa cells were maintained in RPMI 1640 medium containing 10% FBS in a 37 °C incubator with 5% CO_2_. Huh7 cells and A549 cells were maintained in DMEM medium containing 10% FBS in a 37 °C incubator with 5% CO_2_. Cell transfection was performed using Lipofectamine 2000 (Invitrogen, CA, USA) following the manufacturer’s protocol; details are provided in Additional file 1: Experimental Procedures. To generate stable cell lines, HeLa cells were transfected with pcDNA3/EGFP, pcDNA3/EGFP-miR-21 (1× perfect), or pcDNA3/EGFP-CXCR4 (4× bulged) reporter constructs, and neomycin-resistant clones were tested for their ability to yield an EGFP-positive phenotype. For the reporter plasmid carrying EGFP under the regulation of miR-21, the repression of EGFP could be reversed when endogenous miR-21 was blocked by an anti-miR-21 oligonucleotide.

### Co-immunoprecipitation and immunofluorescence

Details are provided in Additional file 1: Experimental Procedures.

### Western blotting

Total protein from HeLa cells transfected with either siRNA or plasmids was extracted using RIPA buffer (1 mM MgCl_2_, 10 mM Tris–HCl pH 7.4, 0.1% SDS, 1% NP-40), and protein expression was analyzed by western blotting. GAPDH served as a loading control. Bands were quantified with Labworks 4.0 software.

### Preparation of cytoplasmic and nuclear fractions

The subfractionation of transfected cells into nuclear and cytoplasmic extracts was performed as previously described [30]. Details are provided in Additional file 1: Experimental Procedures.

### Isolation of total RNA and qRT-PCR

Total RNA was extracted from transfected HeLa cells using Tri-Reagent (Sigma-Aldrich, MO, USA). RNAs were reverse transcribed into cDNA using M-MLV Reverse Transcriptase (TaKaRa, Madison, WI) as specified by the manufacturer. Details are provided in Additional file 1: Experimental Procedures.

### In vivo ubiquitination assay

Details are provided in Additional file 1: Experimental Procedures.

### EGFP fluorescence assay

Details are provided in Additional file 1: Experimental Procedures.

### Statistics

Statistical significance was determined using a two-tailed homoscedastic Student’s *t*-test. For all data analyzed, values were expressed as the mean ± standard deviation (SD), and *P* < 0.05 was considered to be significant. The data generated were representative of at least three separate experiments.

## Results

### Identification of *AGO2* interaction proteins

**Fig. 1:**
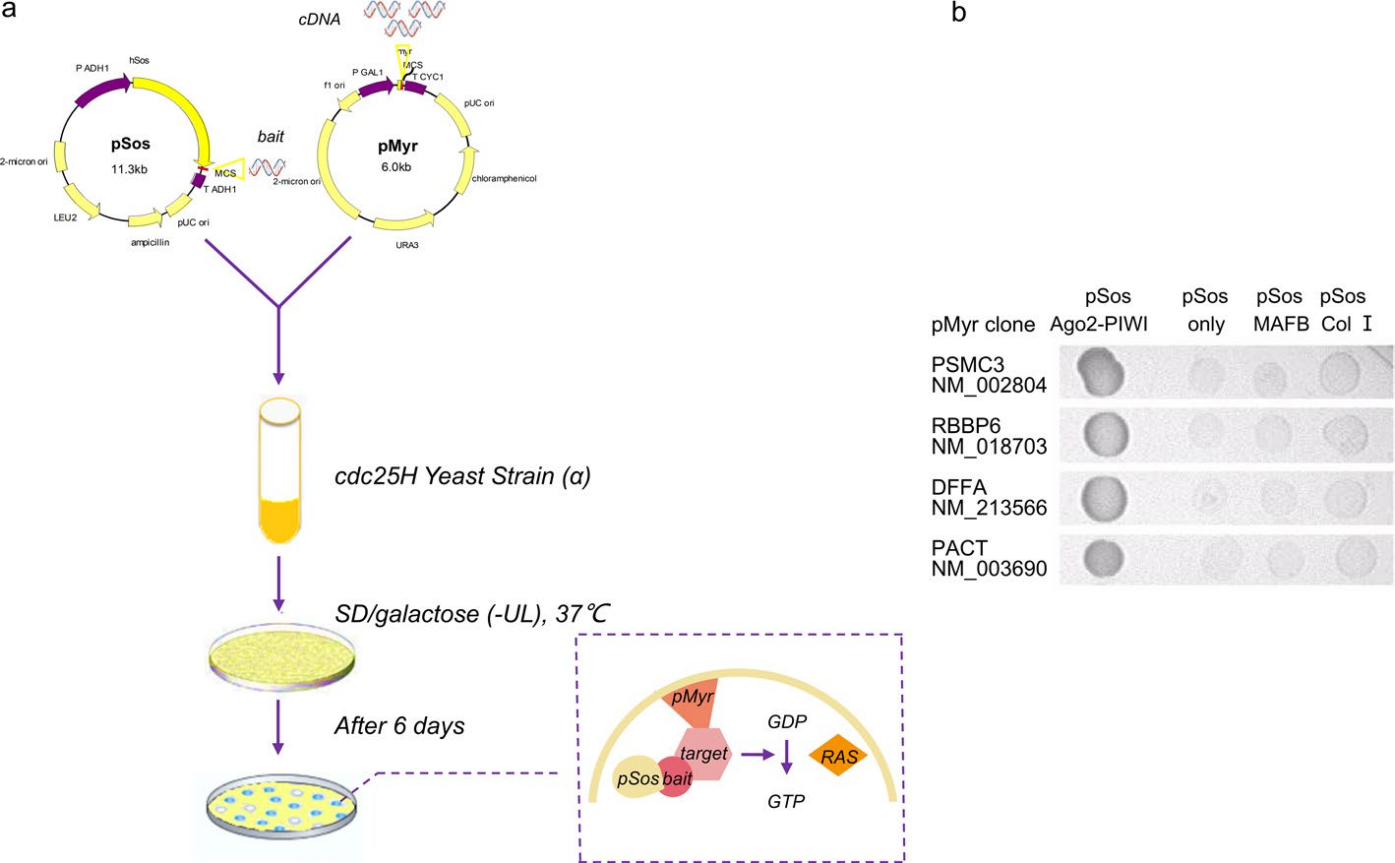
Four proteins interacting with AGO2 were screened and two promote RNAi. **a** Schematic of yeast two-hybrid system used to identify the AGO2 interaction proteins. **b** The interactive clones isolated from a human lung cDNA library were reconfirmed by cotransfection into naive cdc25H yeast with pSOS AGO2-PIWI or the following negative control plasmids: pSOS vector (containing the human SOS gene only), pSOS-MAFB (fusion of the hSOS gene and the transcription factor MAFB) and pSOS-Col I (fusion of the hSOS gene and type IV collagenase). No interaction was detected when the interactive clones were cotransfected with the control plasmids. Accession number of each positive gene is shown

To identify novel proteins associated with human *AGO2*, we employed the CytoTrap two-hybrid system to screen a human lung cDNA library using the *AGO2* PIWI domain as bait (Fig. 1a). This system performed better than conventional GAL4 and LexA two-hybrid systems in assaying interactions in the cytoplasm [31], the main site of *AGO2* localization. We selected the positive interacting colonies on the basis of their ability to grow in appropriate selection medium at 37 °C, and their inserts were sequenced (Fig. 1b). After alignment, four interacting proteins were identified as proteasome 26S subunit, ATPase 3 (*PSMC3*), retinoblastoma binding protein 6 (*RBBP6*), DNA fragmentation factor subunit alpha (*DFFA*), and protein activator of the interferon induced protein kinase (*PACT*), which has been reported to physically interact with* AGO2* [32].

### The coiled-coil region of the N-terminus of *PSMC3* interacts with *AGO2* in an RNA-independent manner

**Fig. 2:**
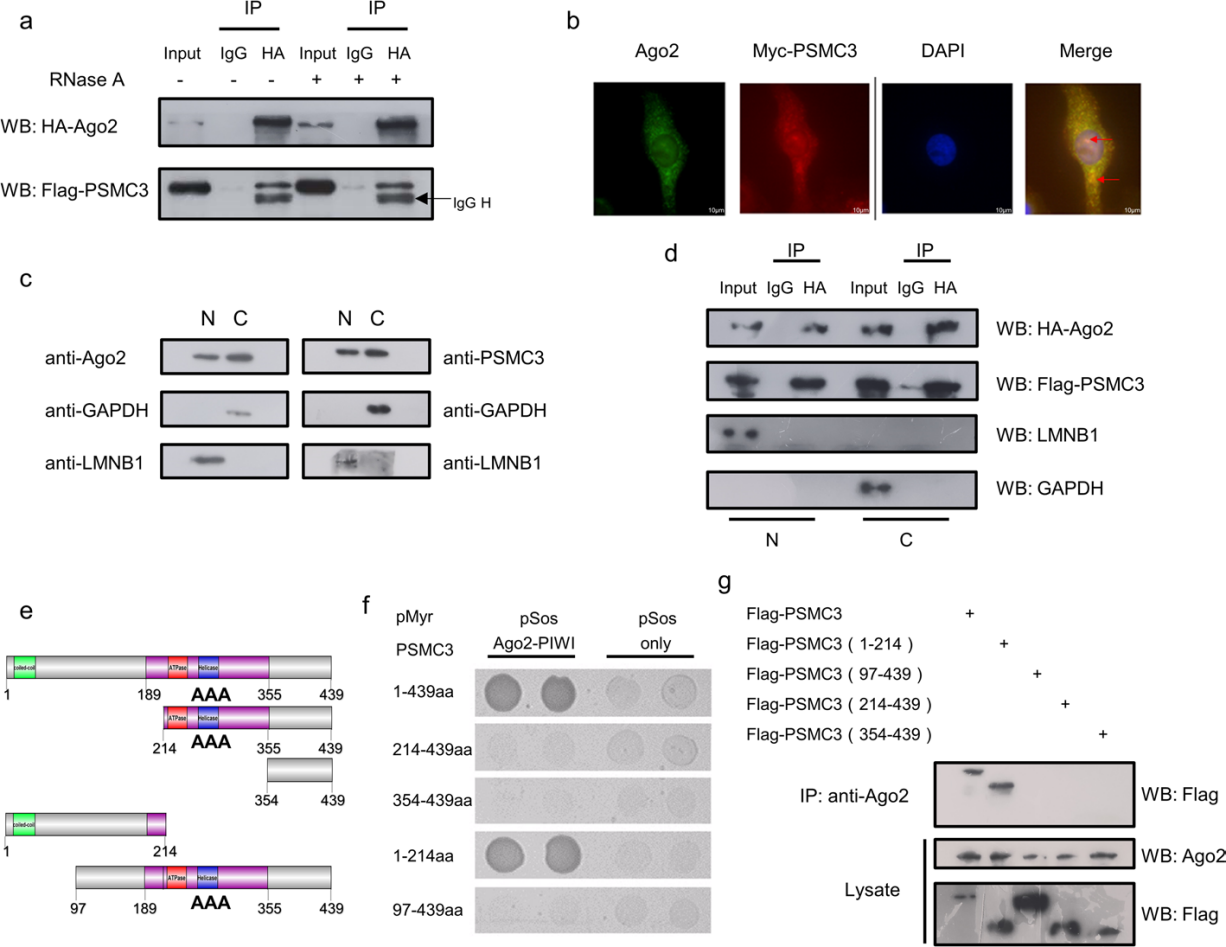
The coiled-coil region of the N-terminus of PSMC3 interacts with human AGO2. **a** PSMC3 interacts with AGO2 in an RNA-independent manner. HeLa cells were co-transfected with HA-AGO2-PIWI and Flag-PSMC3. Immunoprecipitation assays were performed with anti-HA antibody and western blotting with anti-Flag and anti-HA antibodies. For lanes 4, 5, and 6, cell lysates were incubated at room temperature with RNase A (10 mg/ml) for 30 min. **b** PSMC3 colocalizes with AGO2 in HeLa cells. Myc-PSMC3 was transfected into HeLa cells. Forty-eight hours after transfection, immunofluorescence assays were performed with the indicated antibodies, then imaged by confocal microscopy. Scale bar, 10 µm. **c** Western blotting assays were used to detect AGO2 and PSMC3 protein levels in nucleoplasm or cytoplasm from HeLa cells. **d** PSMC3 interacts with AGO2 in both nucleoplasm and cytoplasm. HeLa cells were co-transfected with HA-AGO2 -PIWI and Flag-PSMC3. Then nucleoplasmic or cytoplasmic extracts were harvested and analyzed by western blotting assays. **e** Schematic representation of the full-length and truncated PSMC3s used to map AGO2-binding sites. PSMC3 possesses an N-terminal domain and an AAA ATPase domain that contains an ATP-binding motif (black box) and a helicase motif (white box). **f** A yeast two-hybrid system was used to determine whether pSOS-AGO2-PIWI could interact with the full-length PSMC3 protein or with deletion mutants PSMC3 (214–439), PSMC3 (354–439), PSMC3 (1–214), and PSMC3 (97–439). **g** Mapping the AGO2-interacting site in PSMC3. HeLa cells were transfected with the indicated plasmids encoding Flag-tagged full-length PSMC3 or PSMC3 deletion mutants. After 48 h, cell lysates were immunoprecipitated with anti-AGO2 antibody and then western blotting was performed with anti-Flag and anti-AGO2 antibodies. All results are representative of three independent experiments

Current studies have shown that some proteins interact with* AGO2* in an RNA-dependent manner, while some are RNA independent, which leads to different functions [27, 33–35]. To determine the specific interaction of *AGO2* with *PSMC3* and whether this interaction is dependent on RNA, we treated the lysates using RNase A and then performed co-IP with the ectopically expressed proteins in HeLa cells (Fig. 2a). Our result demonstrates that *PSMC3* interacts with *AGO2* in an RNA-independent manner. To further validate the specificity of this interaction, we also performed co-IP in A549 and Huh7 cells (Additional file 1: Fig. S1a, b), with the same results. Immunofluorescence detection was performed to validate the subcellular co-localization of* PSMC3* and *AGO2*. As shown in Fig. 2b, *PSMC3* was primarily localized with *AGO2* in the cytoplasm, and there appeared to be minor co-localization in the nucleus. Some studies have shown the presence of *PSMC3* and *AGO2* in both cytoplasm and nucleus [29, 36–40]. To clarify whether this interaction exists in both compartments, we first validated their respective cellular localization with nucleoplasmic isolation (Fig. 2c) and immunofluorescence assays (Additional file 1: Fig. S1c), and the results were in accordance with the reports. Then we isolated nucleic or cytoplasmic extracts and performed co-IP assays that confirmed the interaction is present in both compartments (Fig. 2d). *PSMC3* is known to possess an N-terminal coiled-coil domain and a highly conserved AAA (ATPases Associated with a wide variety of cellular Activities) superfamily ATPase domain, which contains a nucleotide-binding motif (ATP-binding site) and a helicase motif [28, 36, 41]. To identify the minimal region of *PSMC3 *necessary for the *PSMC3–AGO2* interaction, full-length and specific-length *PSMC3* cDNAs were tested against the* AGO2* PIWI domain by yeast two-hybrid assay. The N-terminal portion of *PSMC3* was found to be required for the interaction (Fig. 2e, f). Co-immunoprecipitation assays further confirmed this result (Fig. 2g). Coiled-coil domains are commonly involved in protein–protein interactions, and the coiled-coil portion of *PSMC3* has been reported to be involved in interactions with* p14*^*ARF*^ [36]. Here, our results indicate that the coiled-coil region of the N-terminus of *PSMC3 *is required for binding to *AGO2*.

### *PSMC3* is required for efficient RNAi

**Fig. 3:**
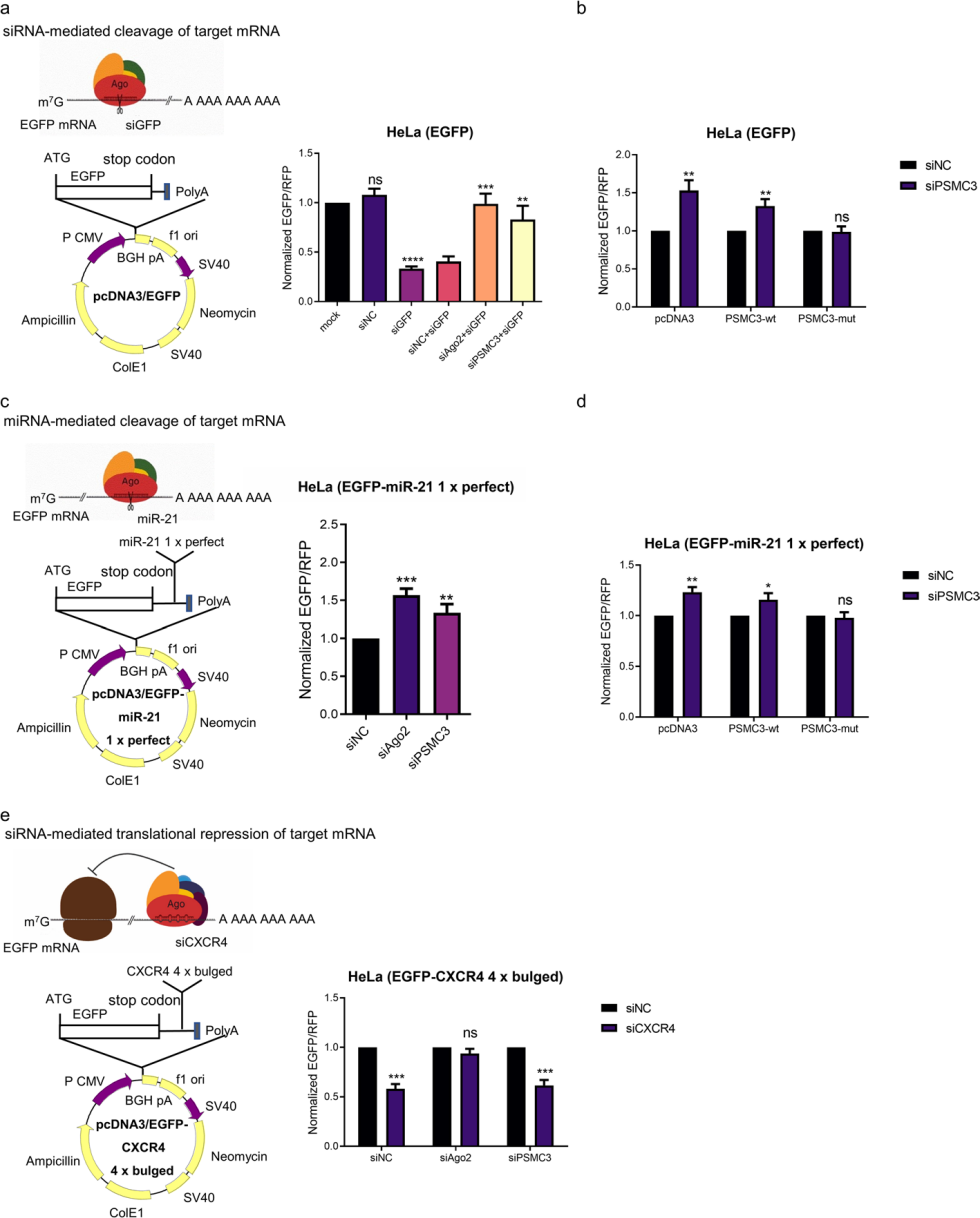
PSMC3 is required for efficient RNAi. **a** Depletion of PSMC3 abolishes the siRNA-mediated cleavage of EGFP mRNA. Overview of the siRNA-mediated cleavage of target mRNA (left). A stable HeLa cell line expressing EGFP was either untransfected or transfected with the indicated siRNAs. pDsRed2-N1, a plasmid expressing RFP (red fluorescent protein), was also included for normalization. After 48 h, expression ratios between the EGFP and RFP reporters were calculated on an F-4500 fluorescence spectrophotometer (right). **b** Expression of the siRNA-resistant PSMC3 mutant overrides the effect of PSMC3 depletion on RNAi. EGFP-expressed HeLa cells were transfected with siNC or siPSMC3, together with pcDNA3 plasmid only or vectors expressing wild-type (wt) or siRNA-resistant (mut) PSMC3. After 24 h, cells were re-transfected with EGFP siRNA. The fluorescence value in the siNC treatment was set to 1. **c** PSMC3 is required for miR-21-mediated mRNA cleavage. Schematic of the miRNA-mediated cleavage of target mRNA (left). A stable HeLa cell line expressing EGFP-miR-21 (which contains a sequence with 1× perfect complementarity to miR-21 in its 3′ UTR) was transfected with the indicated siRNAs. The ratio of EGFP to RFP was normalized to quantify the effect of depleting AGO2 and PSMC3 on RNAi (right). **d** Expression of siRNA-resistant PSMC3 mutant rescues the cleavage activity of miRISC. EGFP-miR-21-expressing HeLa cells were transfected with control or PSMC3 siRNAs together with pcDNA3 plasmid only or vectors expressing wild-type (wt) or siRNA-resistant mutant (mut) PSMC3. The fluorescence value was detected on an F-4500 fluorescence spectrophotometer. **e** Depletion of PSMC3 has no effect on translational repression. Diagram of the siRNA-mediated translational repression of target mRNA (left). A stable HeLa cell line expressing EGFP-CXCR4 (which contains a sequence with 4× bulged CXCR4 binding sites in its 3′ UTR) was transfected with siRNAs targeting AGO2 or PSMC3. After 24 h, cells were re-transfected with control siRNA or CXCR4 siRNA. EGFP protein levels were measured and normalized to RFP as a control (right). In all statistical comparisons, three independent experiments were performed (mean ± SD, *n* = 3, Student’s *t*-test). *, *P* < 0.05, **, *P* < 0.01, ***, *P* < 0.001, ****, *P* < 0.0001

To determine whether *PSMC3* is involved in RNAi pathway, an EGFP fluorescence assay was performed to assess the effect of *PSMC3* on siRNA-induced specific target RNA cleavage, in which the expression of exogenous EGFP was knocked down by specific siRNA (Additional file 1: Fig. S2a). As shown in Fig. 3a, depletion of AGO2 (Additional file 1: Fig. S3a, b) completely abolished RNAi activity, which is consistent with previous reports [6, 42]. The levels of EGFP protein were increased in *PSMC3*-depleted (Additional file 1: Fig. S3c) cells relative to control cells (Fig. 3a and Additional file 1: Fig. S2b, c), indicating that *PSMC3* knockdown reduced siRNA-induced siRISC activity. However, the expression of siRNA-resistant *PSMC3* mutant (Additional file 1: Fig. S3d) rescued this siRISC activity (Fig. 3b). To determine the functional role of *PSMC3* in miRNA-mediated gene silencing, an EGFP-based positive readout reporter assay was applied as previously described [23, 43, 44]. When reporter plasmid pcDNA3/EGFP-miR-21 (1× perfect), which encodes EGFP mRNA containing a fully complementary miR-21 sequence, was transfected into HeLa cells, we observed low EGFP levels due to the effects of endogenous miR-21. However, this repression of EGFP could be reversed by transfection with anti-miR-21 oligonucleotides (Additional file 1: Fig. S2d). Consistent with the knockdown of *AGO2*, depletion of *PSMC3* also relieved endogenous miR-21-mediated repression of the EGFP reporter (Fig. 3c; Additional file 1: Fig. S2d–e). Expression of the siRNA-resistant *PSMC3* mutant overrode the effect of *PSMC3* knockdown on miRNA-induced siRISC cleavage activity (Fig. 3d). These results demonstrate that *PSMC3* is specifically required for small RNA-induced mRNA cleavage in HeLa cells.

We next used a chemokine (C-X-C motif) receptor 4 (*CXCR4*) reporter system [45–47] to determine whether the absence of* PSMC3* impacts cleavage-independent repression. Four bulged *CXCR4* siRNA target sites were introduced into the 3′ UTR of the EGFP reporter gene. HeLa cells stably expressing pcDNA3/EGFP-*CXCR4* (4× bulged) were transfected with the indicated siRNAs. As shown in Fig. 3e and Additional file 1: Fig. S2f, g, silencing of *AGO2* led to a significant derepression of the siRNA reporters, whereas *PSMC3* depletion had no effect. Taken together, our results demonstrate that *PSMC3* depletion results in the inhibition of small-RNA-mediated mRNAs cleavage but not cleavage-independent translation suppression.

### *PSMC3* regulates the stability of cytoplasmic *AGO2* protein at a posttranslational level

**Fig. 4:**
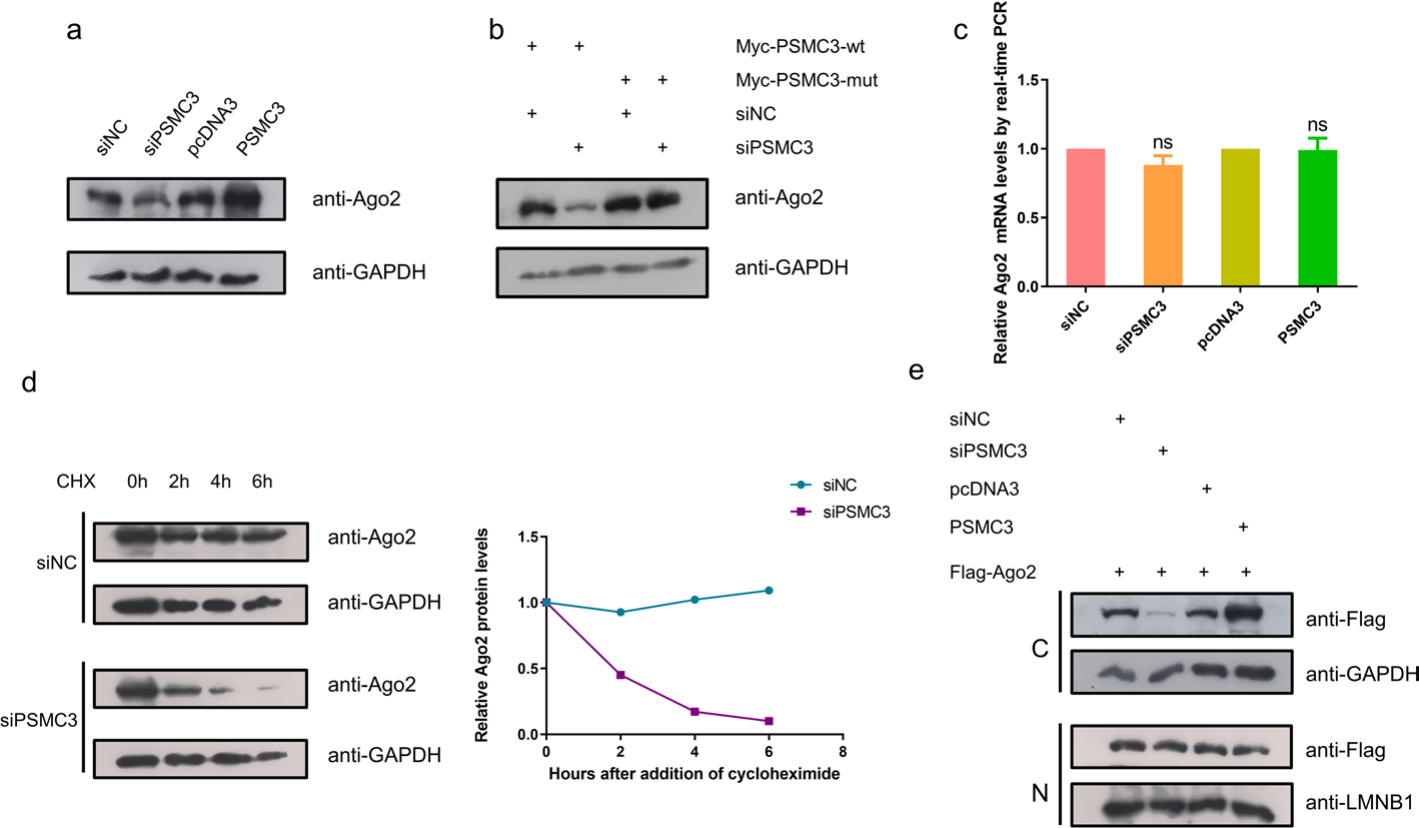
PSMC3 is essential to maintain AGO2 protein levels. **a** PSMC3 increases AGO2 protein levels. HeLa cells were transfected with the indicated plasmids. Extracts were collected at 48 h post-transfection and subjected to western blotting analysis with anti-AGO2 and anti-GAPDH antibodies. **b** Expression of siRNA-resistant PSMC3 abrogates the suppression of AGO2 caused by PSMC3-specific depletion. HeLa cells were co-transfected with plasmids encoding either the wild-type (wt) or mutant (mut) PSMC3 along with siPSMC3 or control siRNA. After 48 h, lysates were analyzed by western blotting with anti-AGO2 and anti-GAPDH antibodies. **c** Real-time RT-PCR analysis of endogenous AGO2 mRNA was performed using total RNA isolated from HeLa cells after 48 h of transfection with the indicated plasmids. GAPDH mRNAs served as the control. In all statistical comparisons, three independent experiments were performed (mean ± SD, *n* = 3, Student’s *t*-test). **d** Depletion of PSMC3 increases AGO2 protein turnover. HeLa cells were transfected with control or PSMC3 siRNA. After 48 h, the cells were treated with 100 µg/mL of cycloheximide (CHX) for the indicated periods and then harvested for immunoblotting with anti-AGO2 and anti-GAPDH antibodies (left). The results were plotted after quantitation (right). **e** Depletion of PSMC3 decreases the amount of AGO2 in the cytoplasm. HeLa cells were co-transfected with the indicated plasmids. Then nucleoplasmic or cytoplasmic extracts were harvested and analyzed by western blotting assays with anti-Flag, anti-GAPDH (cytoplasmic marker), and anti-LMNB1 (nucleoplasmic marker) antibodies. All results are representative of three independent experiments

It has been investigated that *PSMC3* can regulate the amounts of some specific proteins at the posttranslational level [29, 36]; we therefore hypothesized that* PSMC3* may regulate the expression of *AGO2*. So, we examined the effect of *PSMC3* on the levels of *AGO2* in HeLa cells by western blotting. The results showed that *AGO2* protein levels decreased in *PSMC3*-depleted cells and increased in *PSMC3*-overexpressed cells relative to the control (Fig. 4a). To further confirm that *PSMC3* could directly modulate the abundance of *AGO2*, wild-type *PSMC3* and the *PSMC3* mutant were co-transfected into HeLa cells with the indicated siRNAs. The expression of the siRNA-resistant *PSMC3* mutant overrode the suppression of *AGO2* caused by knockdown of the wild-type *PSMC3* (Fig. 4b).

To further determine at which level *PSMC3* affects the* AGO2* protein concentration, we tested whether this was due to the changes in transcription or the stability of the *AGO2* mRNA. RT-qPCR showed that *PSMC3* does not influence the *AGO2* mRNA levels (Fig. 4c). This result suggests that *PSMC3* modulates the amount of *AGO2* at post-translational level. We then performed a cycloheximide assay to determine whether the decline in *AGO2 *levels in *PSMC3*-depleted cells was caused by an increase of *AGO2* protein turnover. As shown in Fig. 4d, knockdown of *PSMC3* shortened the half-life of the *AGO2* protein in HeLa cells, and *AGO2* decayed at higher rates during the time course relative to the control.

Given the interaction of *AGO2* with *PSMC3* in both the cytoplasm and the nucleus (Fig. 2d), we next performed a nucleoplasmic isolation assay. The results demonstrated that cytoplasmic *AGO2* was changed to a greater extent than nuclear *AGO2*, indicating that *PSMC3* primarily regulates the stability of* AGO2* protein in the cytoplasm (Fig. 4e). Immunofluorescence analysis also showed the same result (Additional file 1: Fig. S4a). Meanwhile, we also examined the effect of *AGO2* on *PSMC3* protein levels and found that it did not affect *PSMC3* protein levels (Additional file 1: Fig. S4b). These results suggest that *PSMC3* regulates the stability of cytoplasmic *AGO2* protein at posttranslational level.

### Depletion of *PSMC3* results in reduced *USP14* deubiquitination of *AGO2*

**Fig. 5:**
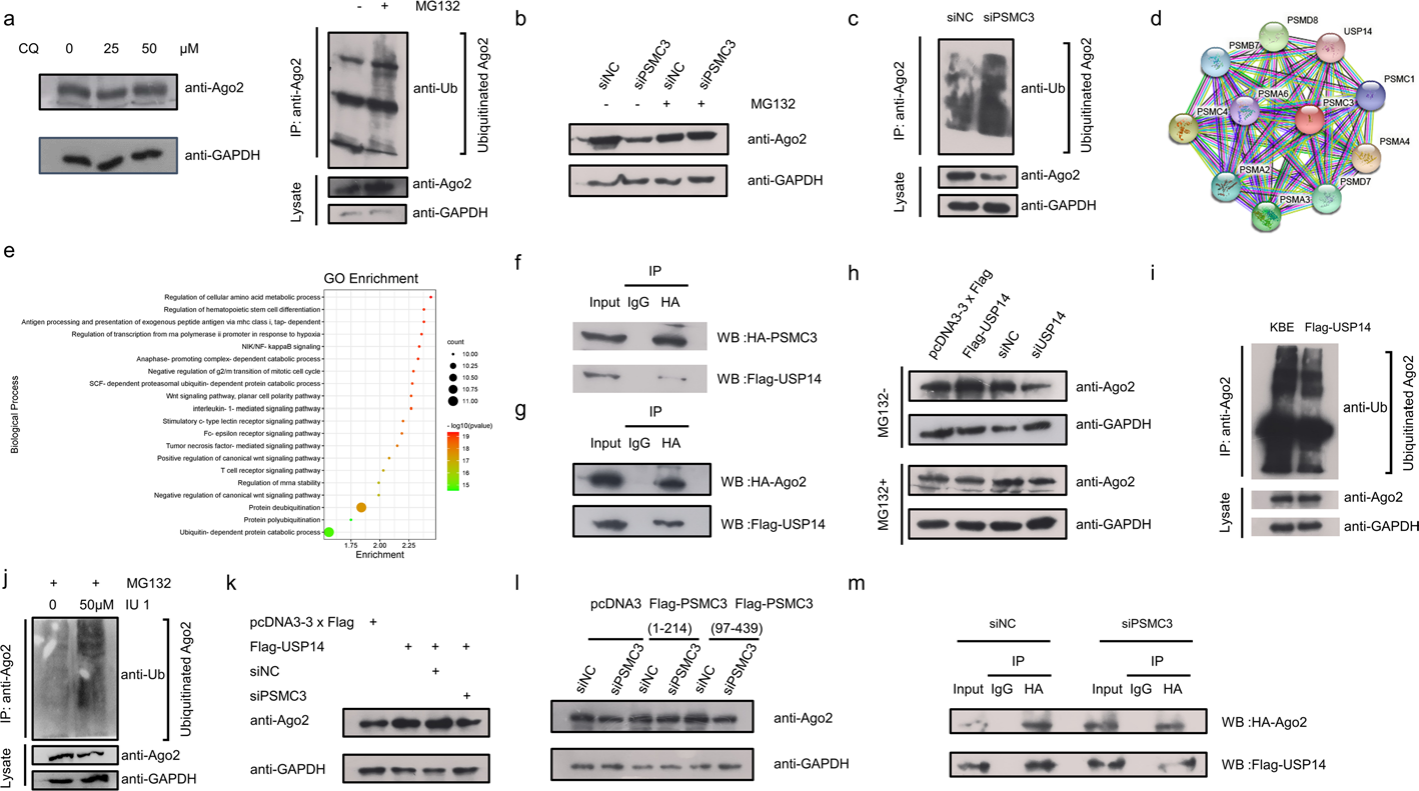
Depletion of PSMC3 inhibits USP14 upregulation of AGO2 proteins. **a** AGO2 degradation via proteasome pathway. After Hela cells were treated with the indicated doses of chloroquine for 18 h, AGO2 protein levels were measured by western blotting assays (left). HeLa cells were incubated with the proteasome inhibitor MG132 (30 µM) or with DMSO for 10 h. Whole-cell lysates were immunoprecipitated with anti-AGO2 antibody and analyzed with anti-ubiquitin, anti-GAPDH, and anti-AGO2 antibodies (right). **b**,**c** Depletion of PSMC3 facilitates AGO2 ubiquitination. **b** HeLa cells were transfected with the indicated plasmids. After 36 h, cells were treated with or without 30 µM MG132 for 10 h. AGO2 protein levels were analyzed by western blotting assays. **c** HeLa cells were transfected with control or PSMC3 siRNAs. After 36 h, cells were incubated with MG132 (30 µM) for 10 h. Whole-cell lysates were immunoprecipitated with anti-AGO2 antibody and analyzed with anti-ubiquitin, anti-GAPDH, and anti-AGO2 antibodies. **d** The PPI network analysis of PSMC3 by the STRING database. **e** The top 20 biological process (BP) terms in the enrichment analysis of the PSMC3-interacting proteins. **f**,**g** PSMC3 (**f**) and AGO2 (**g**) both interact with USP14. HeLa cells were co-transfected with the indicated plasmids. Immunoprecipitation assays were performed with anti-HA antibody and western blotting with anti-Flag antibody. **h**,**i** AGO2 is deubiquitinated by USP14. **h** HeLa cells were transfected with the indicated plasmids. After 36 h, cells were treated with (bottom) or without (top) 30 µM MG132 for 10 h. AGO2 protein levels were analyzed by western blotting assays. **i** HeLa cells were transfected with the indicated plasmids. After 36 h, cells were incubated with MG132 (30 µM) for 10 h. Whole-cell lysates were immunoprecipitated with anti-AGO2 antibody and analyzed with anti-ubiquitin, anti-GAPDH, and anti-AGO2 antibodies. **j** HeLa cells were treated with or without IU1 (an inhibitor of the enzymatic activity of USP14) and MG132 (30 µM) for 10 h. Whole-cell lysates were immunoprecipitated with anti-AGO2 antibody and analyzed with anti-ubiquitin, anti-GAPDH, and anti-AGO2 antibodies. **k** Depletion of PSMC3 inhibits USP14 upregulation of AGO2 proteins. HeLa cells were transfected with the indicated plasmids and then AGO2 protein levels were analyzed by western blotting assays after 48 h. **l** HeLa cells were transfected with either the pcDNA3 vector only or the indicated Flag-tagged PSMC3 (1–214) and siRNA-resistant PSMC3 (97–439) mutant. At 24 h after transfection, cells were split into two aliquots and were retransfected with control or PSMC3 siRNA. Cell lysates were analyzed by western blotting with anti-AGO2 and anti-GAPDH antibodies. **m** The interaction between AGO2 and USP14 is diminished in PSMC3-depletion cells. HeLa cells were cotransfected with the indicated plasmids. Immunoprecipitation assays were performed with anti-HA antibody and western blotting with anti-Flag antibody. All results are representative of three independent experiments

It has been reported that the* AGO2* protein can be posttranslationally modified: modifications such as hydroxylation, phosphorylation, and ubiquitination influence *AGO2* stability [48–52]. Rybak et al. demonstrated that *AGO2* is ubiquitinated by the E3 ubiquitin ligase mLin41 (mouse homolog of lin41) [50]. So, we examined the degradation pathway of *AGO2* protein by using the lysosomal inhibitor chloroquine or the proteasome inhibitor MG132. Upon treatment with MG132 but not chloroquine, high-molecular-weight forms of *AGO2* accumulated (Fig. 5a), indicating that *AGO2* was ubiquitinated in vivo. To further identify the ubiquitination sites of *AGO2*, two ubiquitin site prediction algorithms, BDM-PUB (http://bdmpub.biocuckoo.org/prediction.php) and UbPred (http://www.ubpred.org/) were used, which predicted that the C-terminal region of *AGO2* contains five lysine residues that are potential targets for ubiquitination (Additional file 1: Fig. S5a). To investigate the role of these residues in the ubiquitin-dependent degradation of* AGO2*, single and multiple mutations of lysine to arginine (K-to-R) were introduced into the amino acid sequence of *AGO2* (Additional file 1: Fig. S5b). MG132 treatment resulted in the increased ubiquitination of ectopically expressed HA-tagged* AGO2* relative to DMSO treatment. While single (K726R) or double (K607/608R, K820/844R) lysine residue mutations had little effect on *AGO2* ubiquitination, a decline in ubiquitin-linked *AGO2* was observed with the 4KR mutant, which contained K-to-R changes at positions 607, 608, 820, and 844. Mutations of all five lysine residues (5KR) showed no significant difference when compared with 4KR (Additional file 1: Fig. S5c), indicating that lysine residue 726 is not a ubiquitination site in *AGO2*.

To determine whether *PSMC3* depletion could promote *AGO2* ubiquitination, *PSMC3*-specific or control siRNAs were transfected into HeLa cells with or without MG132. Consistent with our previous results, the knockdown of *PSMC3* reduced *AGO2* protein levels (Fig. 4a). However, when treated with MG132, this effect was eliminated (Fig. 5b). Ubiquitination assays (Fig. 5c) were similarly used to corroborate this result, suggesting that *PSMC3* is involved in regulating the ubiquitin-mediated degradation of *AGO2*. Furthermore, the 4KR and 5KR mutants were completely resistant to degradation following *PSMC3* depletion (Additional file 1: Fig. S5d). All these results indicate that *PSMC3* depletion promotes ubiquitination of C-terminal lysine residues of *AGO2* and, subsequently, the degradation of *AGO2* in the cytoplasm by the proteasome.

To further explore the molecular mechanism by which *PSMC3* regulates *AGO2 *ubiquitination, we predicted the PPI (Protein-Protein Interaction) network of *PSMC3* by the STRING database (http://www.string-db.org) (Fig. 5d). By performing a Gene Ontology (GO) enrichment analysis of the biological processes of these interacting proteins (Fig. 5e), the deubiquitinating enzyme *USP14* attracted our attention, and a literature on a human deubiquitinating enzyme interaction network also supports our prediction[53]. *USP14 *is associated with proteasome regulatory particle and function in chain removal or editing of ubiquitinated proteasome substrates [54, 55]. Therefore, we hypothesized that *PSMC3* acts in stabilizing the *AGO2* protein by *USP14*. Co-IP confirmed that both* PSMC3* and *AGO2* interacted with *USP14* (Fig. 5f, g), while the immunofluorescence assays indicated their colocalization in the cytoplasm (Additional file 1: Fig. S6a, b). We also constructed the truncated plasmids of *USP14* (Additional file 1: Fig. S6c) and defined the interaction sites of *PSMC3* and* AGO2* on *USP14* by co-IP (Additional file 1: Fig. S6d, e). We then tested the effect of *USP14* on *AGO2* protein levels, as well as on ubiquitination levels. It was found that *USP14* could upregulate the protein levels of *AGO2*, and that this effect was offset when treated with MG132 (Fig. 5h). Meanwhile, ubiquitination assays showed that* USP14* inhibited the ubiquitination of *AGO2* (Fig. 5i). It has been reported that *USP14 *can regulate the proteasome activity in a manner independent of its deubiquitylase activity, which in turn affects the stability of some proteins [56]. To determine whether the deubiquitination of *AGO2* by *USP14* is dependent on its deubiquitylase activity, we examined the ubiquitination level of *AGO2* protein after treating HeLa cells with IU1, an inhibitor of the enzymatic activity of *USP14*. The result showed that when the deubiquitylase activity of *USP14* was inhibited, the ubiquitinated *AGO2* protein was accumulated in the cells (Fig. 5j). This suggests that *USP14* regulates the ubiquitination level of the *AGO2* protein in a manner dependent on its deubiquitylase activity. To further elucidate whether the regulation of *AGO2* by *USP14* is dependent on *PSMC3*, HeLa cells were transfected with the indicated plasmids and then *AGO2* protein levels were analyzed by western blotting assays. We found that this upregulation of *USP14* on *AGO2* was relieved upon *PSMC3* knockdown (Fig. 5k). On the basis of the present results and our finding that the effect of* PSMC3 *on *AGO2* stability is dependent on their interaction (Fig. 5l), we speculated that the role of *PSMC3* might be to act as a bridge for* AGO2* to bind to* USP14*. We then examined the binding of *AGO2* to* USP14* in *PSMC3*-depleted cells and found that their interaction was attenuated when *PSMC3* was knocked down (Fig. 5m). Taken together, our results suggest that *USP14* can deubiquitinate *AGO2*, and that this effect is dependent on* PSMC3* as a bridge.

Given that inhibition of proteasome function led to the restoration of* AGO2* expression in* PSMC3*-knockdown cells, we proposed that treatment of cells with the proteasome inhibitor MG132 could abrogate the effect of *PSMC3* depletion on siRISC cleavage activities. We tested this hypothesis in either EGFP or EGFP-miR-21 (1× perfect) reporter systems and observed no significant difference in the siRISC activities of PSMC3-depleted and nondepleted cells upon the addition of MG132 (Additional file 1: Fig. S7a, b). Thus, MG132 treatment can rescue both siRNA and miRNA-guided siRISC cleavage activity in *PSMC3*-knockdown cells.

## Discussion

Although small RNAs and *AGO2* constitute the core endonucleolytic component of the RISC, other proteins might influence the RNA-induced gene silencing pathway through modifying or regulating *AGO2* function. Herein we isolated *PSMC3* as a novel *AGO2*-interacting protein in a yeast two-hybrid screen using the PIWI domain of *AGO2* as bait, and N-terminal part of *PSMC3* containing the coiled-coil motif is responsible for the interaction with *AGO2*. Further results showed that they interact in both nucleoplasm and cytoplasm in an RNA-independent manner. Moreover, our results also suggest that *PSMC3 *may promote RNAi and the functional evidence does support a role of *PSMC3* in small RNA-induced mRNA cleavage by using the reporter systems. However, depletion of *PSMC3* had no significant effect on translation suppression of reporter genes.

So how does *PSMC3* regulate *AGO2* and in turn affect RNAi? *PSMC3* is a multifunctional protein.* PSMC3* (also called HIV Tat-binding protein-1, *TBP-1*) was originally isolated as a protein that binds to the human immunodeficiency virus type 1 (*HIV-1*) Tat transactivator and suppresses Tat-mediated transactivation [57]. It has been shown to regulate transcription, and the transcriptional activity of *PSMC3* is dependent on its conserved nucleotide-binding motif and helicase domain [28]. In addition, *PSMC3* could also control the amounts of proteins at post-translational level. As a component of the regulatory subunit of the 26S proteasome, *PSMC3* seems not to have general effects on proteasome function but rather seems only to affect the stability of specific protein targets. Pollice et al. found that the interaction between *PSMC3* and *p14*^*ARF*^ protects the human oncosuppressor* p14*^*ARF*^ from ubiquitin-independent proteasomal degradation [29, 36]. Therefore, we speculate that *PSMC3* may affect the protein stability of *AGO2*.

In this study, we demonstrate that *PSMC3* regulates stability of cytoplasmic *AGO2* protein at posttranslational level. In *PSMC3*-depleted HeLa cells, the protein level of *AGO2* were reduced, while its mRNA level was not correspondingly decreased. And overexpression of the *PSMC3* N-terminus containing the coiled-coil motif overrode the decrease of* AGO2* level, indicating the effect of *PSMC3* on *AGO2* protein amounts strictly requires their physical interaction. Treatment with cycloheximide demonstrated that the absence of *PSMC3* shortened the half-life of *AGO2* protein, and an increase of *AGO2* protein turnover resulted in a decline of cytoplasmic *AGO2* protein in HeLa cells.

There is some controversy about the degradation pathway of *AGO2*. Rybak et al. found that the E3 ubiquitin ligase mLin41 can mediate the ubiquitination of *AGO2* [50]. While Martinez et al. demonstrated that *AGO2* protein is degraded in lysosomes in mouse embryonic cells [58]. Our results suggest that *AGO2* is degraded via the proteasome pathway and there are four ubiquitination sites present at the C-terminus of AGO2: K607, K608, K820, and K844. We thus hypothesized that *PSMC3* maintains the stability of *AGO2* by preventing its ubiquitination. The results showed that depletion of *PSMC3* enhanced *AGO2* ubiquitination, leading to *AGO2* degradation through the 26S proteasome. But what is the mechanism by which* PSMC3* prevents *AGO2* ubiquitination? Are other proteins involved in this process?

The STRING database predicted* USP14* that may interact with *PSMC3*. *USP14* is associated with proteasome regulatory particle and function in chain removal or editing of ubiquitinated proteasome substrates [54, 55]. Loss of *USP14* in mammalian cells or *Ubp6* in yeast results in increased degradation of ubiquitinated proteins and reduction of free ubiquitin molecules, indicating that *USP14* is required for the recycling of ubiquitin molecules [59, 60]. Prion proteins can be regulated by *USP14*, and intracellular prion protein levels are reduced when *USP14* catalytic activity is inhibited [61]. Our results demonstrated that* PSMC3* and *AGO2* both interact with *USP14* in cytoplasm and *USP14* can regulate the deubiquitination of *AGO2* through its deubiquitinase activity. Moreover, this effect requires the involvement of *PSMC3*, indicating *PSMC3* acts in stabilizing the* AGO2* protein by *USP14*.

**Fig. 6:**
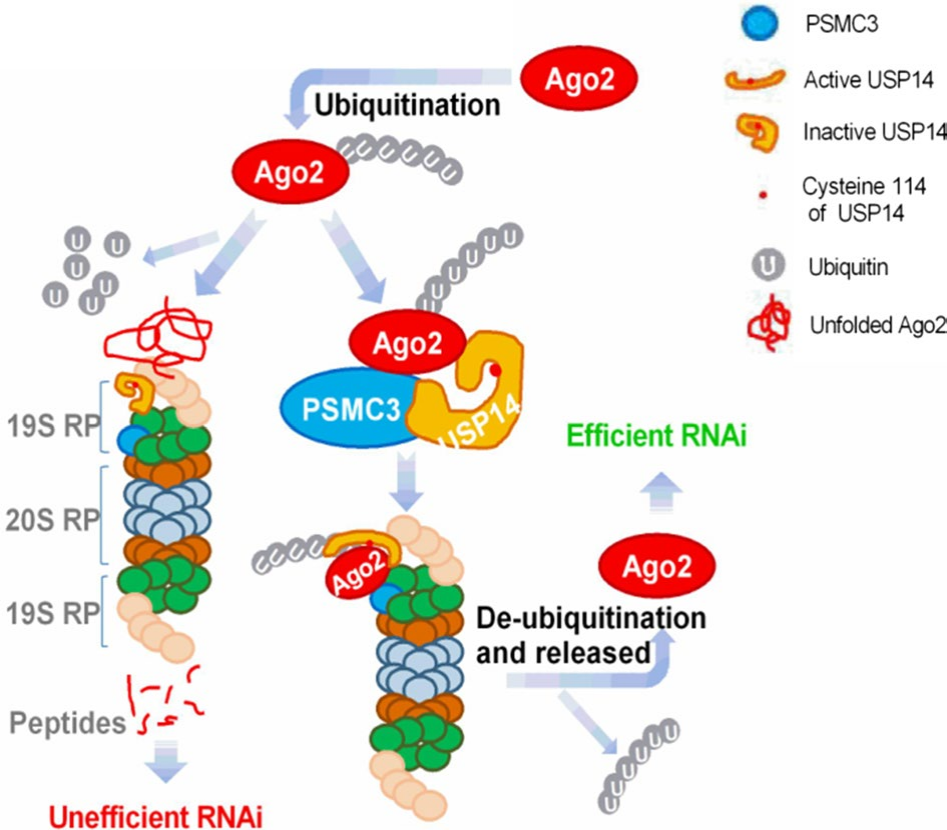
A model for PSMC3 function in AGO2-dependent RNAi. The ubiquitinated AGO2 protein is recruited to the proteasome 19S regulatory subunit by interacting with PSMC3 and then interacts with USP14, which binds 26S proteasome reversibly to form AGO2–PSMC3–USP14 complex. Thereafter, PSMC3 assists with USP14 in exposing its active center and promotes USP14 deubiquitination of AGO2 so that AGO2 proteins can be released from the 26S proteasome and function in an effective RNAi. Otherwise, the ubiquitinated AGO2 is degraded in the 20S catalytic subunit after defolding and displacement

The present study provides a model for *PSMC3* function in *AGO2*-dependent RNAi as shown in Fig. 6. The ubiquitinated *AGO2* protein is recruited to the proteasome 19S regulatory subunit by interacting with *PSMC3* and then interacts with *USP14*, which binds 26S proteasome reversibly to form *AGO2–PSMC3–USP14* complex. Thereafter, *PSMC3* assists with *USP14* in exposing its active center and promotes* USP14*-mediated deubiquitination of *AGO2* so that *AGO2* proteins can be released from the 26S proteasome and function in an effective RNAi. Otherwise, the ubiquitinated *AGO2* is degraded in the 20S catalytic subunit after defolding and displacement. On the basis of this model, it will be of interest to investigate how changes in *PSMC3* levels correlate with *AGO2* levels during physiological and pathological processes.

However, there are still many unanswered questions. Regarding the fact that *PSMC3 *depletion affects siRISC activity but not miRISC activity, the same is true for Qi et al. Their results have shown that type I collagen prolyl-4-hydroxylase [*C-P4H(I)*] physically interacts with* AGO2* and catalyzes proline hydroxylation of the *AGO2* protein. Depletion of *C-P4H(I)* subunits reduced the stability of *AGO2* resulting in impairment of small-RNA-programmed siRISC activity, but miRISC activity was not altered upon *C-P4H(I)* knockdown[49]. Since the only protein involved in targeted cleavage is *AGO2* and all AGOs can be involved in translational repression, we speculate that this result may be due to the compensatory effect of other AGO proteins. For *PSMC3* affecting the protein levels of cytoplasmic *AGO2* rather than nuclear *AGO2*, we found that *USP14* was not localized in the nucleus through the Uniprot database (https://www.uniprot.org/), and our results confirmed this (Additional file 1: Fig. S8). However, these are just our speculations, and more studies are needed to test these hypotheses.

## Conclusion

Our findings demonstrate that *PSMC3 *plays an important role in assisting *USP14* to deubiquitinate *AGO2*, which in turn maintains the stability of *AGO2* to ensure effective RNAi.

## Supplementary information

**Additional file 1**: **FigureS1**. PSMC3 interacts with AGO2. **Figure S2**. PSMC3 is required for mRNA cleavage rather than translational repression. **Figure S3**. Depletion of PSMC3 by siRNAs. **FigureS4**. PSMC3 is essential to maintain AGO2 protein levels. **Figure S5**.Determination of the AGO2 ubiquitination sites. **Figure S6**. PSMC3 and AGO2 both interact with USP14. **Figure S7**. Proteasome inhibition abrogates the effect of PSMC3 depletion on siRISC activities. **Figure S8**. USP14 protein is localized in cytoplasm.

## Data Availability

All relevant data supporting the findings of this study are available in the article, Additional files, or from the corresponding authors on reasonable request. BDM-PUB is a web site for protein ubiquitination sites prediction (http://bdmpub.biocuckoo.org/prediction.php). UbPred is a web site for protein ubiquitination sites prediction (http://www.ubpred.org/). STRING is a database for protein association networks analysis (http://www.string-db.org). UniProt is a database of protein information (http://www.uniprot.org/)

## Abbreviations

AAA: ATPases Associated with a wide variety of cellular Activities
AGO2: Argonaute 2
C-P4H(I): Type I collagen prolyl-4-hydroxylase
CXCR4: Chemokine (C–X–C motif) receptor 4
DFFA: DNA fragmentation factor subunit alpha
GO: Gene Ontology
HIV-1: Human immunodeficiency virus type 1
PACT: Protein activator of the interferon induced protein kinase
PPI: Protein–protein interaction
PSMC3: Proteasome 26S subunit, ATPase, 3
RBBP6: Retinoblastoma binding protein 6
RNAi: RNA interference
RISC: RNA-induced silencing complex
USP14: Ubiquitin specific peptidase 14
