# Supplementary material for: PSMC3 promotes RNAi by maintaining AGO2 stability through USP14
Source: Cell Mol Biol Lett. 2022 Dec 17;27:111. doi: 10.1186/s11658-022-00411-y (PMC9759854; doi:10.1186/s11658-022-00411-y)
Supplement: Supplementary file 1 — Additional file 1: FigureS1. PSMC3 interacts with AGO2. Figure S2. PSMC3 is required for mRNA cleavage rather than translational repression. Figure S3. Depletion of PSMC3 by siRNAs. FigureS4. PSMC3 is essential to maintain AGO2 protein levels. Figure S5.Determination of the AGO2 ubiquitination sites. Figure S6. PSMC3 and AGO2 both interact with USP14. Figure S7. Proteasome inhibition abrogates the effect of PSMC3 depletion on siRISC activities. Figure S8. USP14 protein is localized in cytoplasm. [file 11658_2022_411_MOESM1_ESM.pdf]

Supplementary Information

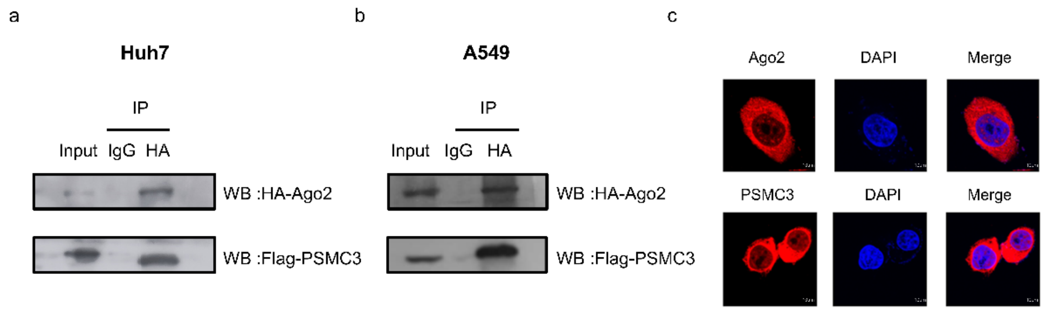

Figure S1. PSMC3 interacts with AGO2

a and b. PSMC3 interacts with AGO2. (a) Huh7 or (b) A549 cells were co-transfected with HA-AGO2 -PIWI and Flag-PSMC3. Immunoprecipitation assays were performed with anti-HA antibody and Western blotting with anti-HA and anti-Flag antibodies. **c.** Immunofluorescence assays were performed to detect the location of AGO2 and PSMC3 in HeLa cells and imaged by confocal microscopy. Scale bar, 10 μm. All results are representative of three independent experiments.

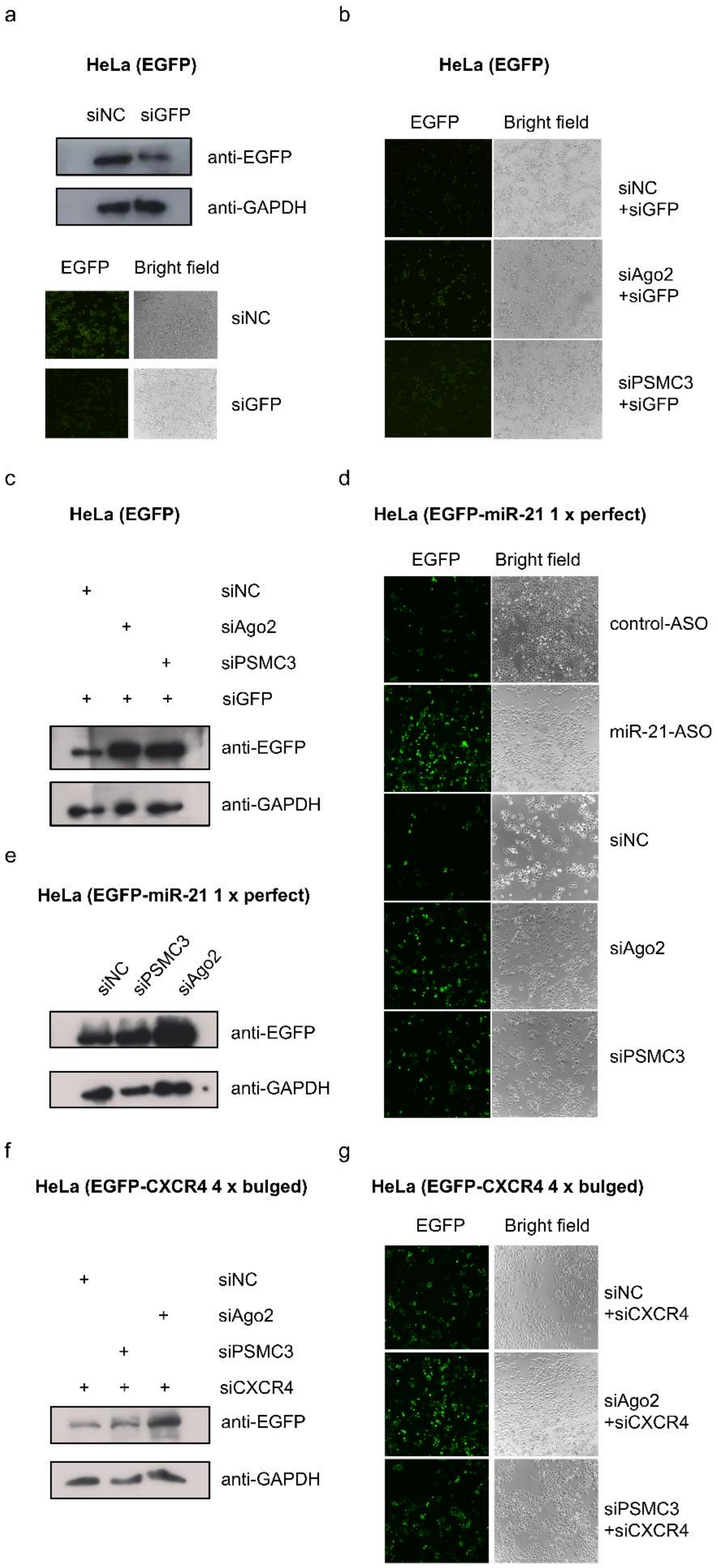
a
HeLa (EGFP)
siNC
siGFP
anti-EGFP
anti-GAPDH
EGFP
Bright field
siNC
siGFP
b
HeLa (EGFP)
EGFP
Bright field
siNC +siGFP
siAgo2 +siGFP
siPSMC3 +siGFP
c
HeLa (EGFP)
siNC
siAgo2
siPSMC3
siGFP
anti-EGFP
anti-GAPDH
d
HeLa (EGFP-miR-21 1 x perfect)
EGFP
Bright field
control-ASO
miR-21-ASO
siNC
siAgo2
siPSMC3
e
HeLa (EGFP-miR-21 1 x perfect)
siNC
siPSMC3
siAgo2
anti-EGFP
anti-GAPDH
f
HeLa (EGFP-CXCR4 4 x bulged)
siNC
siAgo2
siPSMC3
siCXCR4
anti-EGFP
anti-GAPDH
g
HeLa (EGFP-CXCR4 4 x bulged)
EGFP
Bright field
siNC +siCXCR4
siAgo2 +siCXCR4
siPSMC3 +siCXCR4

Figure S2. PSMC3 is required for mRNA cleavage rather than translational repression

a. EGFP-expressing HeLa cells were transfected with control siRNA or siGFP. After 48 hours, digital images (lower panel) were obtained with a fluorescence microscope. Then cells were harvested and analyzed by Western blotting assays (upper panel). **b and c.** Depletion of PSMC3 abolishes the siRNA-mediated cleavage of mRNA. A stable HeLa cell line expressing EGFP was transfected with the indicated siRNAs. After 48 hours, digital images (b) were obtained with a fluorescence microscope. Then cells were harvested and analyzed by Western blotting assays (c). **d and e.** PSMC3 is required for miR-21-induced mRNA cleavage. A stable HeLa cell line expressing EGFP-miR-21 (which contains a sequence with 1 x perfect complementarity to miR-21 in its 3' UTR) was transfected with an miR-21 antisense oligomer or control oligomer as well as control siRNA or siRNAs against AGO2 or PSMC3. After 48 hours, digital images (d) were obtained with a fluorescence microscope. And whole-cell lysates were analyzed by Western blotting assays (e). **f and g.** Depletion of PSMC3 has no effect on translational repression. A stable HeLa cell line expressing EGFP-CXCR4 (which contains a sequence with 4 x bulged CXCR4 binding sites in its 3' UTR) was transfected with the indicated siRNAs. After 48 hours, digital images (g) were obtained with a fluorescence microscope. Then cells were harvested and analyzed by Western blotting assays (f). All results are representative of three independent experiments.

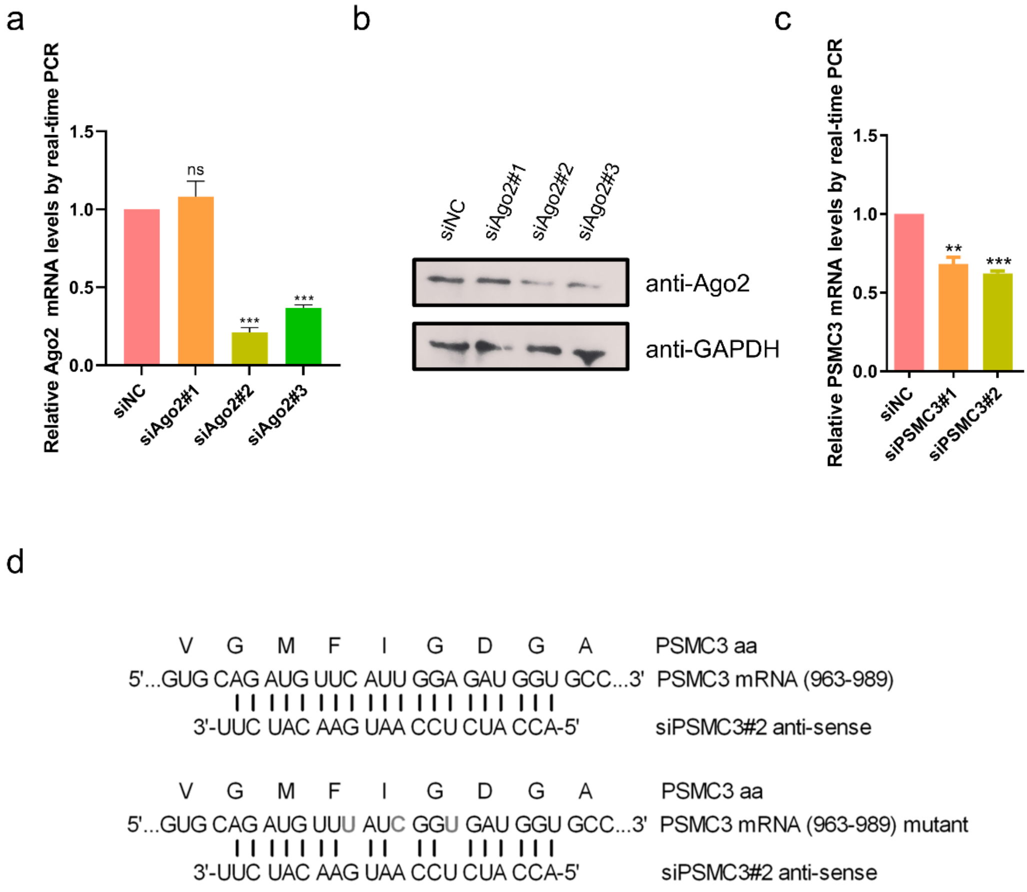

Figure S3. Depletion of PSMC3 by siRNAs

a. HeLa cells were transfected with control siRNA or AGO2 siRNAs (siAGO2#1, siAGO2#2 or siAGO2#3). Real-time RT-PCR analysis of AGO2 mRNA was performed using total RNA isolated from HeLa cells after 48 hours of transfection. In all statistical comparisons, three independent experiments were performed (mean ± S.D., n = 3, Student's t-test). ***, $P < 0.001$. **b.** HeLa cells were co-transfected with the indicated plasmids. Cell lysates were analyzed by Western blotting with anti-AGO2 and anti-GAPDH antibodies. **c.** HeLa cells were transfected with control siRNA or PSMC3 siRNAs (siPSMC3#1 or siPSMC3#2). Real-time RT-PCR analysis of PSMC3 mRNA was performed using total RNA isolated from HeLa cells after 48 hours of transfection. In all statistical comparisons, three independent experiments were performed (mean ± S.D., n = 3, Student's t-test). **, $P < 0.01$, ***, $P < 0.001$. **d.** The region of PSMC3 targeted by siPSMC3#2 is indicated. Three-point mutations were introduced into the siRNA-binding site without affecting the PSMC3 amino acid sequence.

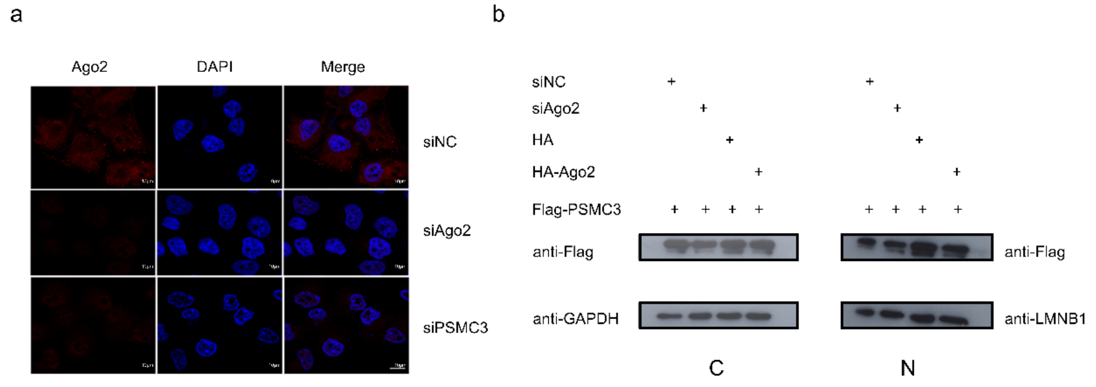

Figure S4. PSMC3 is essential to maintain AGO2 protein levels

a. Depletion of PSMC3 decreases the amount of AGO2 in the cytoplasm. HeLa cells were transfected with control siRNA or siRNA against human AGO2 or PSMC3. At 48 h post-transfection, cells were subjected to immunofluorescent staining with antibody against AGO2. Cells were stained with DAPI to visualize nuclei, and the images were digitally merged. Scale bar, 10 µm. **b.** AGO2 has no effect on PSMC3 protein levels. HeLa cells were co-transfected with the indicated plasmids. Then nucleoplasmic or cytoplasmic extracts were harvested and analyzed by Western blotting assays. All results are representative of three independent experiments.

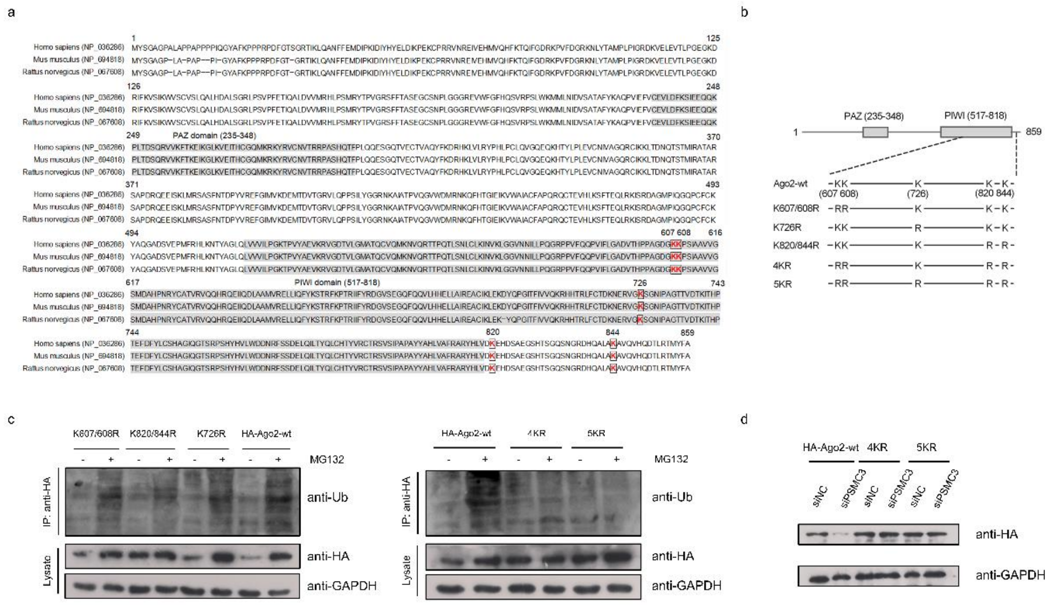

Figure S5. Determination of the AGO2 ubiquitination sites

a. The putative ubiquitination sites in the AGO2 C-terminus were predicted by BDM-PUB and UbPred.

b. Schematic representation of AGO2 mutants with C-terminal K-to-R (lysine residues replaced by arginines) changes. **c.** Multiple C-terminal lysine residues target AGO2 for ubiquitination. HeLa cells were transfected with HA-tagged wild-type (wt) AGO2 or AGO2 with C-terminal K-to-R mutations. After 36 hours, cells were split into two aliquots; one was treated with 30 μM MG132 and one with DMSO for 10 h. Total cell extracts were immunoprecipitated with an anti-HA antibody and probed in Western blotting with anti-ubiquitin antibodies; lysates were also probed for the presence of HA-tagged protein. **d.** Degradation of AGO2 in PSMC3 knockdown cells is diminished by lysine mutations. HeLa cells were transfected with HA-tagged wild-type (wt) or mutant AGO2. At 24 h post-transfection, cells were split into two aliquots and re-transfected with control or PSMC3 siRNA. Cell lysates were analyzed by Western blotting with anti-HA and anti-GAPDH antibodies.

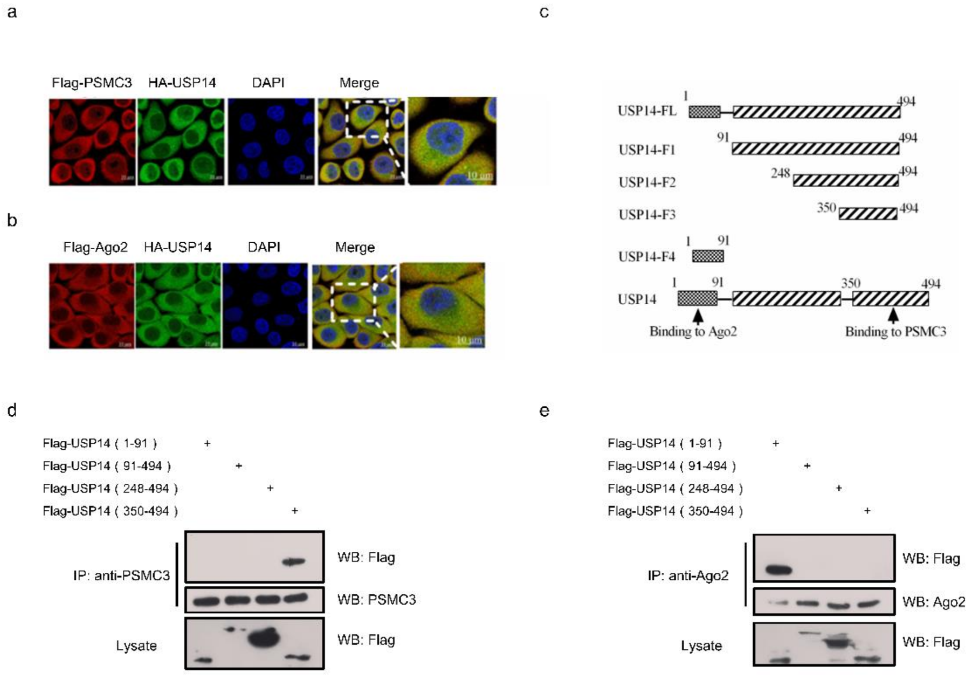

Figure S6. PSMC3 and AGO2 both interact with USP14

a. PSMC3 interacts with USP14. HeLa cells were co-transfected with Flag-PSMC3 and HA-USP14. Immunofluorescence assays were performed with the indicated antibodies, then imaged by confocal microscopy. Scale bar, 10 μm. **b.** AGO2 interacts with USP14. HeLa cells were co-transfected with HA-USP14 and Flag-AGO2. Immunofluorescence assays were performed to detect the co-location of AGO2 and USP14 in HeLa cells and imaged by confocal microscopy. Scale bar, 10 μm. **c.** Schematic representation of the full-length and truncated mutants of USP14 used to map AGO2- and PSMC3-binding sites. **d.** Mapping the PSMC3-interacting site in USP14. HeLa cells were transfected with the

indicated plasmids. After 48 hours, cell lysates were immunoprecipitated with anti-PSMC3 antibody and then Western blotting with anti-PSMC3 and anti-Flag antibodies. **e.** Mapping the AGO2-interacting site in USP14. HeLa cells were transfected with the indicated plasmids. Immunoprecipitation assays were performed with anti-AGO2 antibody and Western blotting with anti-AGO2 and anti-Flag antibodies. All results are representative of three independent experiments.

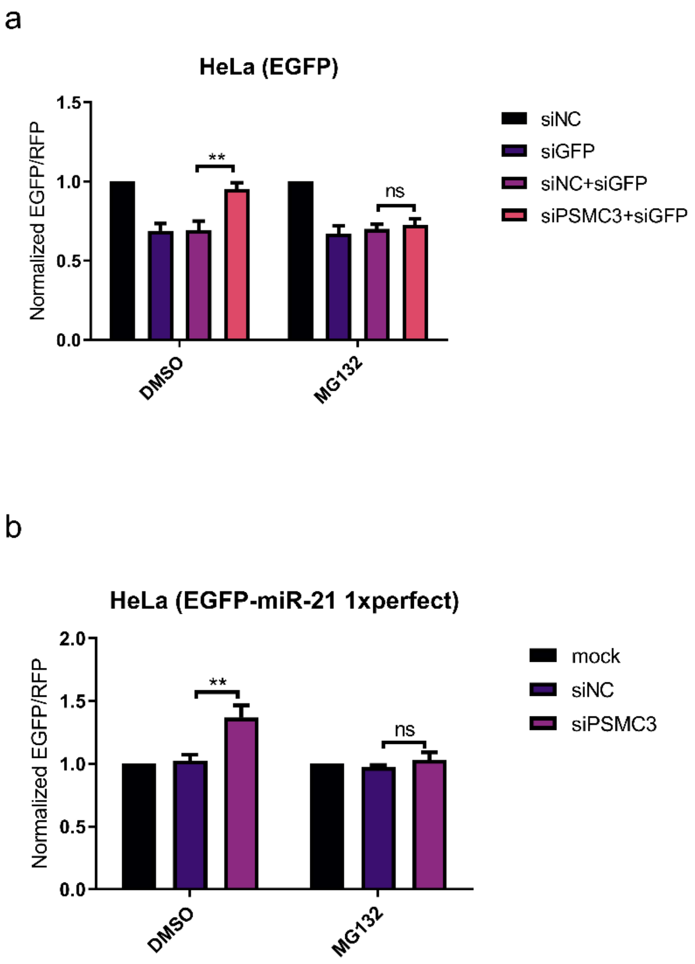

Figure S7. Proteasome inhibition abrogates the effect of PSMC3 depletion on siRISC activities

a. EGFP-expressing HeLa cells were transfected with the indicated siRNAs. pDsRed2-N1, which encodes RFP, was included for normalization. At 48 h after transfection, cells were treated with 30 µM MG132 or DMSO for 10 h, and the ratio of EGFP to RFP was normalized to that observed with siNC treatment. Data are presented as the mean ± standard deviation for three independent experiments. **, $P < 0.01$. **b.** EGFP-miR-21 (1 x perfect)-expressing HeLa cells were either untransfected or transfected

with control or PSMC3 siRNAs. After 48 hours, cells were treated with 30 μM MG132 or DMSO for 10 h. The EGFP value was normalized by the RFP value. Data are presented as the mean ± standard deviation for three independent experiments. **, $P < 0.01$.

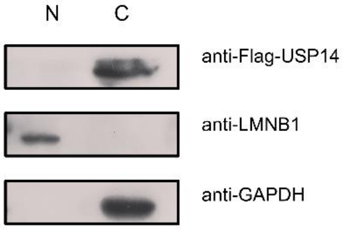

Figure S8. USP14 protein is localized in cytoplasm

Western blotting assays were used to detect USP14 protein levels in nucleoplasm or cytoplasm from HeLa cells.

Supplemental Experimental Procedures

Plasmids and oligos

The coding region of the human AGO2 PIWI domain was cloned in frame with the hSOS domain of pSOS (Stratagene, Santa Clara, CA). To generate HA-tagged AGO2 PIWI, the fragment of the human AGO2 PIWI domain was inserted into a modified pcDNA3 plasmid (Invitrogen, Carlsbad, CA) containing an N-terminal 3 x HA-tag. Full-length AGO2 was amplified by PCR using the primers 5'-GCC ACC GAA TTC GCC ACC ATG TAC TCG GGA GCC-3' and 5'-GCC ACC ATG CGT CGA CGC AGC AAA GTA CAT GGT GCG CAG-3' and cloned into the *Eco*RI and *Sal*I restriction sites of pcDNA3 with a 3 x HA-tag or a 3 x Flag-tag. Multiple K-to-R mutants in AGO2 were generated by site-directed mutagenesis of HA-AGO2 using a PCR-based strategy. To obtain HA-, Myc- and Flag-tagged PSMC3, the full-length open reading frames (ORFs) were cloned into the *Eco*RI and *Xho*I sites of pcDNA3 by PCR using primers 5'-CGG AAT TCA TGA ATC TGC TGC CGA ATA TTG-3' and 5'-CCTGTCTCGAGTAGGCGTAGTATTGTAGGTTGGC-3'. ORF truncations of PSMC3 were constructed by PCR techniques and cloned into the *Eco*RI and *Xho*I sites of the pcDNA3 with a 3 x Flag-tag and pMyr vectors. To generate siRNA-resistant mutations in PSMC3, three-point mutations were introduced into the siRNA binding site of the PSMC3 ORF by PCR using the forward mutagenesis primer 5'-GTG CAG ATG TTT ATC GGT GAT GGT GCC A-3'. To obtain Flag-tagged USP14, the full-length open reading frames (ORFs) were cloned into the *Bam*HI and *Xho*I sites of pcDNA3 by PCR using

primers 5'-CGG AAT TCA TGA ATC TGC TGC CGA ATA TTG-3' and 5'-CCTGTCTCGAGTAGGCGTAGTATTGTAGGTTGGC-3'. ORF truncations of USP14 were constructed by PCR techniques and cloned into the *Bam*HI and *Xho*I sites of the pcDNA3 with a 3 x Flag-tag. Myc-tagged RBBP6, PACT, DFFA was subcloned from the pMyr plasmid obtained from positive clones selected by yeast two-hybrid screen.

The EGFP gene from the pEGFP-N2 vector (Clontech, Mountain View, CA) was subcloned into pcDNA3 and is referred to as pcDNA3/EGFP. Reporter plasmid pcDNA3/EGFP-miR-21 (1 × perfect) was generated by PCR as described previously[44]. The EGFP reporter construct carrying four bulged binding sites for CXCR4 siRNA antisense was subcloned from the pRL-TK CXCR4 4 × plasmid (Addgene, Cambridge, MA) into pcDNA3/EGFP 3' UTR using the *Xba*I and *Apa*I sites. All constructs were checked by sequencing and BLAST searching.

Oligonucleotides complementary to miR-21 were synthesized by IDT (Coralville, IA, USA) as described previously[71]. The sequences of the siRNAs (passenger strand) are as follows: siPSMC3#1, 5'-AAC AAG ACC CUG CCG UAC CdTdT-3'; siPSMC3#2, 5'-GAU GUU CAU UGG AGA UGG UdTdT-3'; siAGO2#2: 5'-GCA CGG AAG UCC AUC UGA AdTdT-3'; siEGFP, 5'-GCA GCA CGA CUU CUU CAA GdTdT-3'; siCXCR4, 5'-GUU UUC ACU CCA GCU AAC ACA-3'; siNC, 5'-UUC UUC GAA CGU GUC ACG UdTdT-3'; siUSP14, 5'-UCA GCA UCG UAA CAC CAG AAG AUA UdTdT-3'. All experiments were performed with siPSMC3#2 and siAGO2#2, except where otherwise indicated.

Yeast two-hybrid screen

A Stratagene Cytotrap system human lung library (La Jolla, CA, US) was screened according to the manufacturer's instructions. The pSOS bait construct (pSOS/AGO2 PIWI) and pMyr target library were co-transformed into *S. cerevisiae* strain cdc25H and plated on synthetic glucose minimal medium lacking leucine and uracil. After 2-4 days at 25°C, replica plates of the transformants were made and transferred to a 37°C incubator for 6 days. Putative positive clones were picked, and yeast plasmid DNA was isolated and transformed along with the pSOS bait construct into yeast for another interaction test. Positive clones were subsequently sequenced, and the identification of each clone was determined by performing BLAST searches against the human genome.

Cell culture and transfection

HeLa cells were inoculated in cell culture plates or cell culture flasks and transfected when the cell density was 50%-80%. Lipofectamine 2000 and plasmid or siRNA were diluted with appropriate amounts of Opti-MEM, respectively. After 5 min, they were mixed and incubated for 20 min. Then the mixture was added to the culture plate or culture flask and replaced with the appropriate volume of cell culture medium after 6 h. The amount of siRNA was 100 nM, and the amount of plasmid was 1μg/ml. The corresponding assays were performed 48 hours after transfection.

Co-immunoprecipitation and immunofluorescence

At 48 h post-transfection, HeLa cells were lysed with buffer containing 20 mM Tris-Cl, pH 7.5, 150 mM NaCl, 1 mM NaF, 0.1% NP-40, 10% glycerol and complete protease inhibitor cocktail (Roche) at 4ºC for 30 min. After centrifugation, 1 ml of the resulting supernatant was incubated with anti-AGO2, anti-HA or anti-PSMC3 antibodies at 4°C for 16 h. The protein-antibody complexes were precipitated by incubating the lysate with Protein A/G UltraLink Resin (Pierce, IL, USA) for 5 h under constant rotation at 4°C. After incubation, the resin was washed four times with lysis buffer, and proteins were eluted with 2×loading buffer, boiled for 5 min, and processed for Western blotting.

For immunofluorescence detection, transfected HeLa cells that had been grown on slides were fixed using 4% paraformaldehyde in 1×PBS for 30 min at 4°C. Permeabilization was performed with 0.2% Triton-X 100 for 5 min at 4°C. Samples were blocked for 30 min in 1×PBS containing 10% (w/v) donkey serum. All primary and secondary antibodies were diluted in 2% donkey serum. Samples were incubated with the anti-AGO2 primary antibody (1:500 dilution), anti-PSMC3 primary antibody (1:500 dilution), anti-Myc primary antibody (1:1000 dilution), anti-HA primary antibody (1:500 dilution) or anti-Flag primary antibody (1:1000 dilution) in a humid chamber overnight at 4°C. After washing with 1×PBS, the slides were incubated with the TRITC/FITC-conjugated secondary antibody (1:200 dilution) for 1 h at 4°C. Finally, the cells were stained with 0.05 g/ml DAPI in PBS for 2 min at RT. Mounted slides were viewed in a confocal microscope, and image analysis was performed using Leica Confocal Software and Adobe Photoshop 7.0 (Adobe Systems, Mountain View, CA).

Preparation of cytoplasmic and nuclear fractions

The subfractionation of transfected cells into nuclear and cytoplasmic extracts was performed as previously described. Briefly, at 48 h post-transfection, 2×10^6 cells were collected by trypsin/EDTA

treatment and suspended in 1×PBS. Cell pellets were lysed by gentle inversion after resuspension in 150 μl of PBS containing 0.4% NP-40, followed by centrifugation at 1,000×g for 3 min. The supernatants (cytoplasmic extract) were removed and placed in fresh tubes. The nuclear pellets were washed once with 100 μl of PBS containing 0.1% NP-40 and resuspended in 100 μl of RIPA buffer (nuclear extract).

Isolation of total RNA and qRT-PCR

Total RNA was extracted from transfected HeLa cells using Tri-Reagent (Sigma-Aldrich, MO, USA). RNAs were reverse transcribed into cDNA using M-MLV Reverse Transcriptase (TaKaRa, Madison, WI) as specified by the manufacturer. All PCRs were performed with the following conditions: 94°C for 4 min followed by 40 cycles of 94°C for 30 s, 58°C for 30 s and 72°C for 30 s. SYBR Premix Ex TaqTM kit (TaKaRa, Madison, WI) was used in standard qPCR reactions according to the manufacturer's instructions, and PCR was performed on an iQ5 Real-Time PCR system (Bio-Rad, Hercules, CA). The primers used are the following sequences: 5'-AGA CCT GTA TGA GAA CCC-3' and 5'-TGT TGC TTT CAC TCT CAG-3' for AGO2; 5'-CGC AAG ATA GAG TTC CCG-3' and 5'-TTA TTG CGC ACT TCA GCC-3' for PSMC3. 5'-CAA AAT GGT GAA GGT CGG TGT-3' and 5'-TGA TGT TAG TGG GGT CTC GCT-3' for GAPDH. Real-time PCR results were analyzed and expressed as relative expression of the CT (threshold cycle) value using the 2(-Delta Delta C(T)) method (Livak and Schmittgen, 2001).

In vivo ubiquitination assay

HeLa cells were transfected with either siRNA or plasmids expressing HA-tagged wild-type AGO2 or AGO2 mutants. At 48 h post-transfection, transfected cells were treated with MG132 or solvent DMSO as a control. After indicated hours, the cells were lysed in buffer (25 mM Tris-Cl pH 7.4, 150 mM NaCl, 0.1% NP-40, 10% glycerol, 1 mM dithiothreitol, 10 mM NaF, 8 mM β-glycerophosphate, 20 mM sodium vanadate, 50 mM chloroacetamide and protease inhibitor cocktail) as previously described55. For immunoprecipitation, cell lysates (3 mg proteins) were incubated with anti-AGO2 or anti-HA antibodies at 4°C for 16 h, then bound to Protein A/G UltraLink Resin (Pierce, IL, USA). Western blot analysis was performed using an anti-ubiquitin antibody.

EGFP fluorescence assay

Stable HeLa cell lines were either transfected with 40 pmol of indicated siRNAs or co-transfected with 40 pmol of siRNA and 0.6 μg of plasmid in 24-well plates. The vector pDsRed2-N1 (Clontech, Mountain View, CA), which encodes RFP, was also included for normalization. At 48 h post-transfection, cells were lysed with RIPA lysis buffer. The intensities of EGFP and RFP fluorescence were detected

with a Fluorescence Spectrophotometer F-4500 (Hitachi, Tokyo, Japan).
